# Characterization of a protozoan Phosducin-like protein-3 (PhLP-3) reveals conserved redox activity

**DOI:** 10.1371/journal.pone.0209699

**Published:** 2018-12-31

**Authors:** Rachel L. Kooistra, Robin David, Ana C. Ruiz, Sean W. Powers, Kyle J. Haselton, Kaitlyn Kiernan, Andrew M. Blagborough, Ligin Solamen, Kenneth W. Olsen, Catherine Putonti, Stefan M. Kanzok

**Affiliations:** 1 Department of Biology, Loyola University Chicago, Chicago, IL, United States of America; 2 Department of Life Sciences, Imperial College London, South Kensington Campus, London, United Kingdom; 3 Bioinformatics Program, Loyola University Chicago, Chicago, IL, United States of America; 4 Department of Chemistry and Biochemistry, Loyola University Chicago, Chicago, IL, United States of America; 5 Department of Computer Science, Loyola University Chicago, Chicago, IL, United States of America; Institut national de la santé et de la recherche médicale - Institut Cochin, FRANCE

## Abstract

We recently identified three novel thioredoxin-like genes in the genome of the protozoan parasite *Plasmodium* that belong to the Phosducin-like family of proteins (PhLP). PhLPs are small cytosolic proteins hypothesized to function in G-protein signaling and protein folding. Although PhLPs are highly conserved in eukaryotes from yeast to mammals, only a few representatives have been experimentally characterized to date. In addition, while PhLPs contain a thioredoxin domain, they lack a CXXC motif, a strong indicator for redox activity, and it is unclear whether members of the PhLP family are enzymatically active. Here, we describe PbPhLP-3 as the first phosducin-like protein of a protozoan organism, *Plasmodium berghei*. Initial transcription analysis revealed continuous low-level expression of *pbphlp-3* throughout the complex *Plasmodium* life cycle. Attempts to knockout *pbphlp-3* in *P*. *berghe*i did not yield live parasites, suggesting an essential role for the gene in *Plasmodium*. We cloned, expressed and purified PbPhLP-3 and determined that the recombinant protein is redox active *in vitro* in a thioredoxin-coupled redox assay. It also has the capacity to reduce the organic compound *tert-Butyl hydroperoxide* (TBHP) *in vitro*, albeit at low efficiency. Sequence analysis, structural modeling, and site-directed mutagenesis revealed a conserved cysteine in the thioredoxin domain to be the redox active residue. Lastly, we provide evidence that recombinant human PhLP-3 exhibits redox activity similar to that of PbPhLP-3 and suggest that redox activity may be conserved in PhLP-3 homologs of other species. Our data provide new insight into the function of PhLP-3, which is hypothesized to act as co-chaperones in the folding and regulation of cytoskeletal proteins. We discuss the potential implications of PhLP-3 as a thioredoxin-target protein and possible links between the cellular redox network and the eukaryotic protein folding machinery.

## Introduction

**Fig 1:**
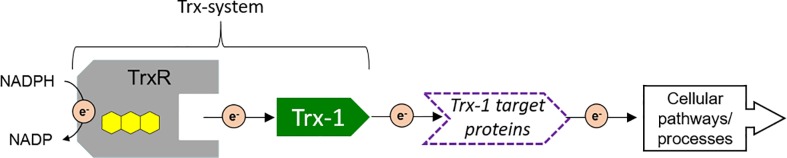
The central role of the thioredoxin system and its target proteins. The flavoenzyme thioredoxin reductase (TrxR) and the small redox-active thioredoxin (Trx-1) constitute the Trx-system, which supplies reducing equivalents to Trx-target proteins that are involved in various cellular processes.

Thioredoxins (Trx) and thioredoxin-like proteins (Tlp) are small ubiquitous proteins present throughout the tree of life. The members of the Trx-superfamily contain at least one Trx-domain, which forms a characteristic compact 3D structure known as the Trx-fold with a central twisted beta-sheet sandwiched by at least three alpha helices [1–3]. Some Trx and Tlps exhibit thiol-based redox activity facilitated by a [-CXXC-] active site motif located in the Trx-domain. A prominent example is the canonical Trx-1, a highly conserved cytosolic protein that contains a [-CGPC-] active site motif and functions as a cellular master redox regulator [3]. As such, Trx-1 has multiple target proteins in a variety of cellular pathways, such as antioxidant defense, nucleotide biosynthesis, transcription factors, and protein folding. For Trx-1 to be active it needs to be in its reduced state (Trx(SH)_2_). Trx-1 is supplied with reducing equivalents by NADPH via the flavoenzyme thioredoxin reductase (TrxR). Together TrxR and Trx-1 form the Trx-system of a cell (Fig 1).

The protozoan parasite *Plasmodium* belongs to the phylum *Apicomplexa* and is the causative agent of the infectious disease Malaria. The parasite is transmitted between people by *Anopheles* mosquitoes. Initial studies of the Trx-system in *Plasmodium* focused on its role in antioxidant defense and parasite survival [4, 5]. One goal of these studies was to identify potential anti-malarial drug targets [6, 7]. Over time, new roles for the Trx-system were described as more Trx and Tlps were identified in the genomes of various *Plasmodium* species [7]. Studies have now expanded from the disease causing blood stages of *Plasmodium* in the human host to the developmental stages of the parasite inside the mosquito vector. Yet, our understanding of *Plasmodium* cell biology in the mosquito is still very limited. *Plasmodium berghei* is a prominent model organism for the study of malaria in mice and in mosquitoes [8].

More than fifty genes in the *P*. *berghei* genome are currently annotated as putative Trx or Tlp due to the presence of a predicted Trx-domain. Those that have been experimentally characterized [6, 7] are implemented in diverse cellular pathways, including nucleotide metabolism, antioxidant defense, redox signaling and red blood cell invasion [9]. We recently determined that three of the predicted Trx-domain containing genes belong to the phosducin family of proteins (PhLP) [10]. PhLPs are highly conserved from yeast to mammals [11, 12] and these *Plasmodium* genes represent the first identified in a protozoan organism [10].

PhLPs represent a family of small Trx-domain containing proteins that exhibit the same basic structural organization consisting of an N-terminal helix domain followed by a Trx-domain and a short C-terminus [11]. Unlike Trx-1, however, PhLPs do not possess the characteristic [CXXC] active site and are therefore considered not to be redox active [13, 14]. The increasing number of PhLPs described in diverse organisms led to the formation of three PhLP-subfamilies organized with respect to sequence homology and putative cell function [13]. Members of the PhLP-1 subfamily are closely related to the canonical *phosducins* (or PDCs), which were originally identified in mammalian retina rod photoreceptor cells where they function in trimeric G-protein signaling [12]. Members of the PhLP-2 and PhLP-3 subfamilies are both hypothesized to play roles as co-chaperones via the formation of a ternary complex with the *chaperonin-containing t-complex polypeptide 1* (CCT) [15]. However, the biochemical properties of these highly conserved proteins remain unclear.

Here we report on the characterization of PbPhLP-3, of the protozoan parasite *P*. *berghei*. We first determined the transcription profile of *pbphlp-3* during parasite development in the mouse host and during early development in the mosquito. We then made an attempt to disrupt the gene in *P*. *berghei*. We cloned, expressed and purified PbPhLP-3 for initial biochemical characterization and tested its potential redox activity *in vitro*. Lastly, based on the high sequence and structural homology between *Plasmodium* and human PhLP-3 (HsPhLP-3; *thioredoxin-domain containing protein 9* (TXNDC9)) we cloned, expressed and purified the HsPhLP-3 and investigated the potential conservation of redox activity within the PhLP-3 subfamily.

## Results

### Organization of the *P*. *berghei* PhLP family

We previously identified three putative Trx-like genes in the genome of the rodent malaria parasite *P*. *berghei* as members of the *phosducin-like* family of proteins (PhLP), specifically PBANKA_1204800, PBANKA_0519700, and PBANKA_1231200 [10, 16]. PBANKA_1204800 was designated as *PhLP-1* as it was the first PhLP we identified in a *Plasmodium* species [10]. However, re-analysis of sequence data from multiple organisms suggests that PBANKA_1204800 belongs to the PhLP-3 subgroup, while PBANKA_1231200 is more closely related to the PhLP-1 subgroup [S1 Fig]. The annotation of PBANKA_0519700 as PhLP-2 was confirmed. Consequently, for the remainder of this report, we will refer to the members of the *P*. *berghei* PhLP family as follows: PBANKA_1231200 as PbPhLP-1, PBANKA_0519700 as PbPhLP-2, and PBANKA_1204800 as PbPhLP-3 (Table 1). Here we report on the characterization of PbPhLP-3, which shows the highest conservation among eukaryotes.

**Table 1:** PhLP in *P*. *berghei*.

*P*. *berghei* PhLP family	PbPhLP-1	PbPhLP-2	PbPhLP-3
**NCBI Accession #**	CDS49513.1	CDS45324.1	SCO63514.1
**PlasmoDB Gene ID**	PBANKA_1231200	PBANKA_0519700	PBANKA_1204800
**Genomic Location (chr:position)**	12: 1204764–1205699	5: 707940–709565	12: 201652–202233
**# of Exons**	1	8	1
**Length (AA)**	311	213	193
***P*. *falciparum* ortholog identity**	64.1%	68.57%	75.13%
**# of Trx Domains**	2	1	1
**Previous Alias/ID**	PbPhLP-3	PbPhLP-2	PbPhLP-1
**Transcriptome [17, 18]**	all stages*	blood stages*	all stages*
**Proteome [17, 19]**	gametocytes	N/A	sporozoites, gametocytes
**Putative function**	N/A	N/A	cell redox homeostasis
**Phenotype**	N/A	non-essential [20]	Essential [this report]

### *pbphlp-3* is constitutively expressed

**Fig 2:**
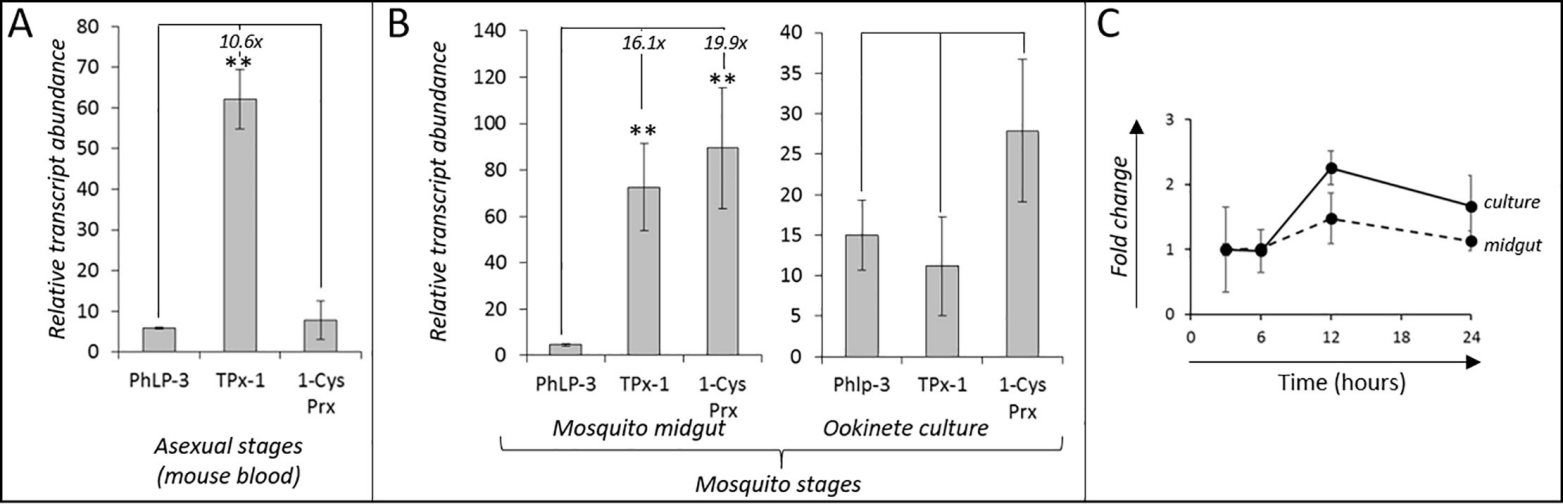
Relative expression of *pbphlp-3* in different parasite stages. Relative transcript abundance of *pbphlp-3* and the control genes *thioredoxin-dependent peroxidase-1* (*tpx-1*) and *1-cys peroxiredoxin* (*1-cys prx*) in **A.** asexual *P*. *berghei* parasites from mouse blood **B.** mosquito stage parasites developing either in the mosquito midgut or under culture condition. Indicated fold changes specify the difference to *pbphlp-3* expression. ** signify statistical significance with a p-value <0.05. **C**. Temporal transcription profile of *pbphlp-3* in developing mosquito-stage parasites during the first 24 hours in mosquito midguts or under culture conditions, respectively. For the investigation of mosquito stage parasites female mosquitoes were allowed to feed on infected mice and blood fed midguts were subsequently dissected at 3, 6, 12, and 24 hours post blood meal. For ookinete cultures, samples were taken at the same time points as the midgut dissections. Error bars represent standard deviation of three independent experiments.

Analysis of transcriptome and proteome data sets on PlasmoDB revealed that phlp-3 is actively transcribed by *P*. *berghei* and other *Plasmodium* species, including the human pathogens *P*. *falciparum* and *P*. *vivax* (Table 1). We first confirmed expression of *pbphlp-3* in *P*. *berghei* utilizing quantitative real time-RT PCR (RT-qPCR). Transcript abundance was determined for *pbphlp-3* (S1 Fig and S2 Fig) and two control genes of the Trx-superfamily, *thioredoxin-dependent peroxiredoxin-1* (*tpx-1;* PBANKA_1302800) and *1-cysteine peroxiredoxin* (*1-cys prx*; PBANKA_122800) [21]. *pbphlp-3* expression in *P*. *berghei* was confirmed in samples taken from infected mouse blood containing mixed asexual stages (Fig 2A). The level of *pbphlp-3* transcription was low and comparable to that of the control gene *1-cys prx*. In comparison, the RNA level of *tpx-1* was significantly higher (10.6 fold), which is due to its antioxidant function during blood stage development of the parasite. The expression of both control genes during parasite development in mammalian blood is consistent with recent reports [21–23].

We next investigated whether *pbphlp-3* expression was maintained during parasite development in the mosquito. To this end, we dissected bloodfed midguts from mosquitoes 12 hours after they were allowed to feed on *P*. *berghei*-infected mice. Our data show that low *pbphlp-3* expression persisted during parasite development in the mosquito (Fig 2B) as both control genes showed significantly higher expression levels, with *pbtpx-1* 16-fold higher, and *1-cys prx* 20-fold higher than the *pbphlp-3* transcript (Fig 2B). The increased expression of the control genes was expected as the parasites responds to the hostile conditions in the mosquito bloodmeal [21]. Consequently, when mosquito-stage parasites were allowed to develop under culture conditions, in the absence of the bloodmeal environment, the control genes were not upregulated and expression levels of all three genes showed no significant differences (Fig 2B). This indicates that the midgut environment in the blood meal has no impact on *pbphlp-3* expression. To test this, we determined *pbphlp-3* transcription profiles in parasites developing within mosquito midguts or in culture. No significant modulation of *pbphlp-3* expression was detected in blood meal derived or in culture-derived parasites of the recorded time (Fig 2C). This indicates that *pbphlp-3* expression is largely independent of external challenges in the mosquito midgut. Taken together, our data suggest continuous but low-level *pbphlp-3* expression in parasites from the mouse blood as well as from bloodfed mosquitoes. No stage-specific or environmentally induced upregulation was observed during the investigated parasite stages.

### *pbphlp-3* is essential for *Plasmodium*

**Fig 3:**
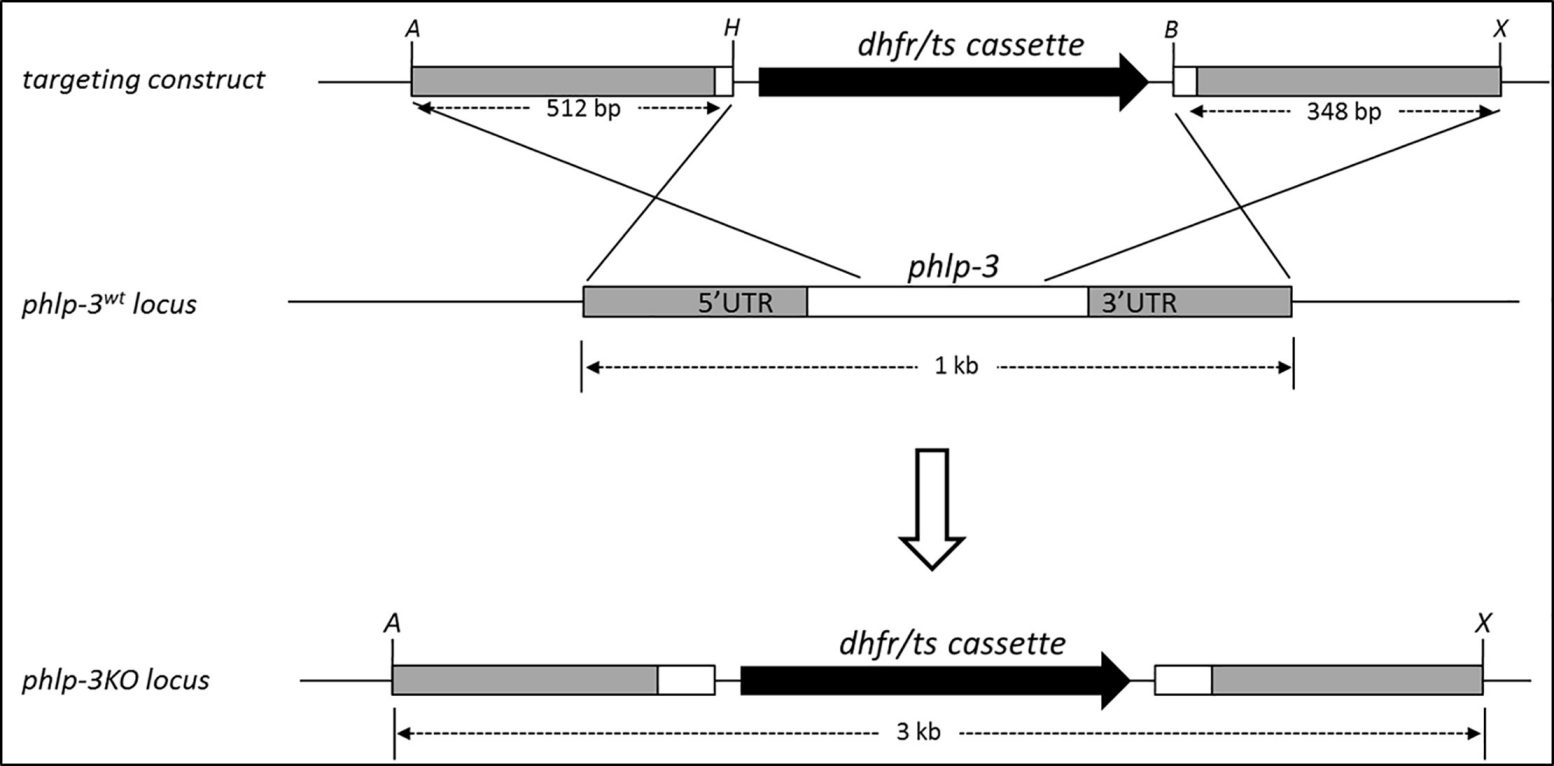
Knockout strategy for the *P*. *berghei phlp-3* locus. To achieve double-crossover target gene disruption a targeting plasmid was constructed in the parent plasmid pBS-DHFR [26]. Two fragments, a 512 bp fragment covering the *pbphlp-3* 5’UTR and a 348 bp fragment corresponding to the 3’UTR were amplified from *P*. *berghei* genomic DNA. The fragments were inserted on each side of the *Toxoplasma gondii* dhfr/ts selection cassette. The linearized plasmid was transfected into purified *P*. *berghei* schizonts following the procedure described in [26, 27].

Our next goal was to knock out *pbphlp-3* in the rodent parasite. *pbphlp-3* is a single-copy, single-exon gene located on chromosome 12 in the *P*. *berghei* genome (Table 1). The open reading frame (orf) spans 582 base pairs and codes for a 193 amino acid protein (S1 Fig). A targeting plasmid was constructed to replace the protein-coding region of *pbphlp-3* with a *T*. *gondii* dhfr/ts selection cassette, which confers to transfected parasites resistance to the antimalarial drug *pyrimethamine* (*pyr*) (Fig 3 and S2 Fig). Following transfection of the plasmid into isolated blood stages of wild-type (WT) *P*. *berghei* the parasites were immediately injected into the tail-veins of naïve mice [24]. Mice were provided with pyr treated drinking water and their blood was frequently checked for drug resistant parasites. A total of four successive trials was conducted. Following each transfection, neither viable *pyr*–resistant integrated parasites, nor viable parasites maintaining *episomes* could be obtained. Positive control transfections performed in parallel successfully generated viable drug-resistant parasites [25]. Based on these results we conclude that *pbphlp-3* is likely to be essential for *P*. *berghei* blood stages.

### Conservation of PhLP-3 sequence, organization, and structure

**Fig 4:**
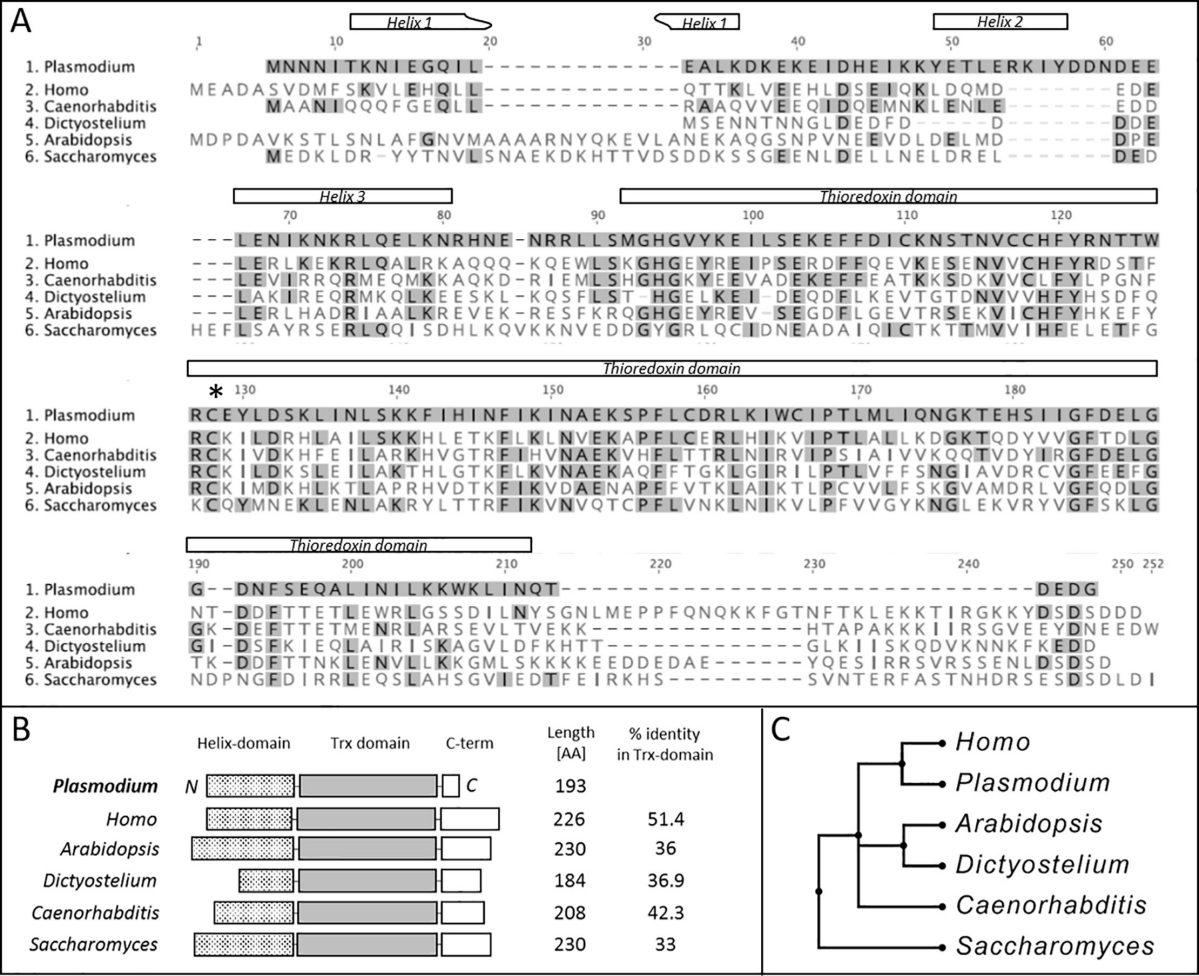
Conservation and organization of PhLP-3 in eukaryotes. **A**. Alignment of *P*. *berghei* with recently characterized PhLP-3 homologs of different species using the MUSCLE algorithm [29]. Amino acids conserved with *P*. *berghei* are highlighted in gray. Predicted domains are indicated above the sequence. The asterisk marks the single conserved cysteine residue. **B**. Comparison of structural PhLP-3 organization between different eukaryotes. Inset table shows percent AA identities within thioredoxin domains. **C**. Maximum likelihood tree of PhLP-3 sequences with bootstrap replication.

Our failed knockout attempts suggest an important role for *pbphlp-3* in the parasite. We therefore turned our attention to the characterization of the PbPhLP-3 protein. Previous phylogenetic analyses indicates high conservation of PhLP-3 among diverse eukaryotic organisms [10, 28]. Alignment of recently characterized PhLP-3 homologues, ranging from fungi to mammals, coupled with secondary structure prediction, revealed that PbPhLP-3 consists of an N-terminal helix domain, followed by a thioredoxin-domain and a short C-terminus, an overall organization characteristic for members of the PhLP family (Fig 4A and 4B) [11]. Notably, the highest degree of conservation resides within the Trx-domain (Fig 4B). A phylogenetic tree based on the Trx-domains of characterized PhLP homologues revealed a higher degree of conservation between the protozoan parasite and the human PhLP-3 (51%) (HsPhLP-3; alias Trx-Containing Protein 9 (TXNDC9); Gene ID: 10190) than with those of other lower eukaryotes, such as *Saccharomyces* (33%) and *Dictyostelium* (37%) (Fig 4C).

### PbPhLP-3 cloning, expression, purification and redox activity

**Fig 5:**
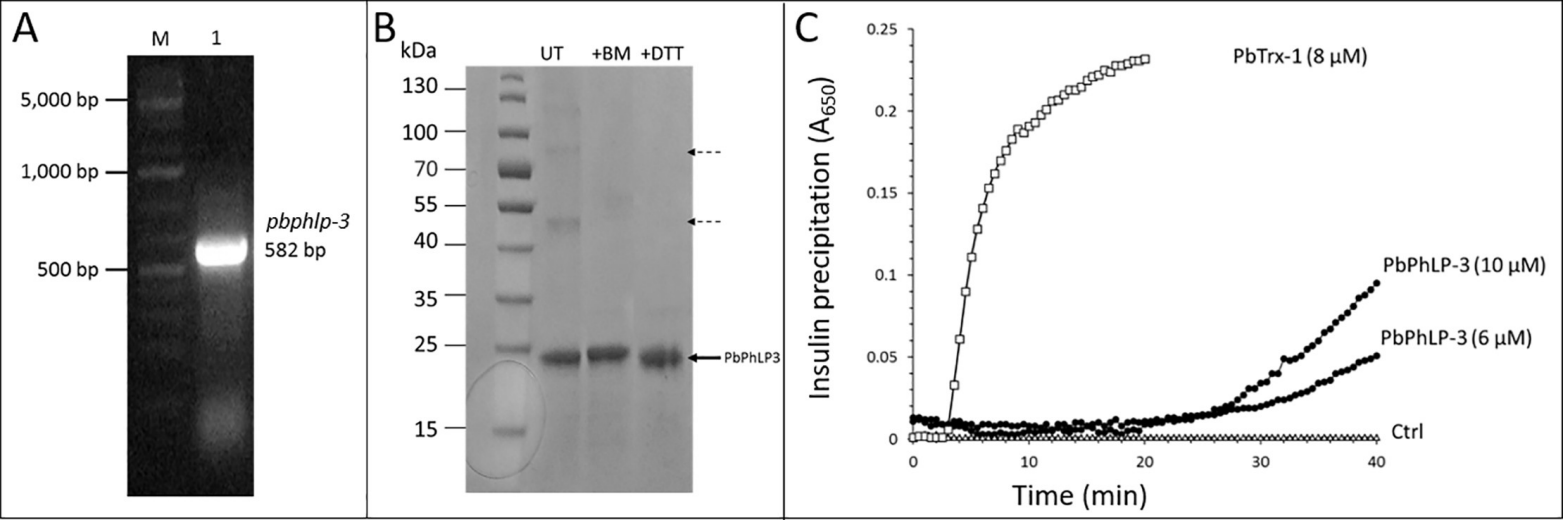
Cloning, recombinant expression and purification of PbPhLP-3. **A**. Agarose gel show amplified *pbphlp-3* orf from *P*. *berghei* cDNA (Lane 1). **B**. SDS Page electrophoresis following recombinant PbPhLP-3 purification over a Ni-NTA column. Samples remained either untreated (UT = untreated) or were pretreated with reducing agents *2-mercaptoethanol* (BM) or *dithiothretiol* (DTT). Solid arrow indicates the recombinant PbPhLP-3 protein incl. HIS-tag. Weak bands in the UT lane (dashed arrows) designate potential multimers of PbPhLP-3. **C**. Insulin-based reduction assay [32] shows precipitation of insulin precipitation in the presence of dithiothreitol (DTT) alone (ctlr) and in the presence of PbTrx-1 or indicated concentrations of PbPhLP-3.

PbPhLP-3 is a 193 AA protein with a calculated molecular weight of 22.9 kDa (Table 1). We generated recombinant PbPhLP-3 with an N-terminal 6xHIS tag (Fig 5A). The recombinant protein was purified using Ni-NTA resin. SDS gel electrophoresis revealed a prominent 24 kDa band, which corresponds to the calculated molecular mass of the tagged PbPhLP-3 (Fig 5B). Additional weaker bands were observed at ~ 50 kDa and ~ 80 kDa, which we hypothesize to represent disulfide-based multimers of PbPhLP-3 as they collapsed in the presence of reducing agents *dithiothreitol* (DTT) and *2-mercaptoethanol* (BM), respectively (Fig 5B).

The absence of a discernable [-CXXC-] active site motif in PhLPs gave rise to the assumption that members of the PhLP-family lack redox activity [13]. Yet, several members of the Trx-superfamily exhibit thiol-based redox activity facilitated by a single cysteine in their respective active sites (monothiol mechanism), e.g. glutaredoxin 5 (Grx5) in yeast [30, 31]. The Trx-domain in PbPhLP-3 contains six cysteines (Fig 4A) raising the possibility that the protein may be redox active. We initially utilized the insulin-reduction assay, which was originally developed as a simple test for investigating the thiol-based redox activity of thioredoxins [32] and serves as an indicator for the presence of redox-active cysteines in Trx and Tlps [6, 33]. It is based on the thiol-dependent reduction of the intermolecular disulfide bridges in insulin by the small electron donor *dithiothreitol* (DTT), which leads to the precipitation of the insulin B-chain. This can be spectrophotometrically measured at 650 nm as an increase in turbidity over time [34]. We used PbTrx-1 as a positive control and show that it effectively catalyzes insulin reduction (Figs 5C and S3A) [32, 35]. Recombinant PbPhLP-3 (6 μM) catalyzed insulin reduction by DTT at an initial rate of 0.0038A min^-1^. Increasing the PbPhLP-3 concentration to 10 μM resulted in a corresponding increase in the insulin reduction-rate by 1.8 fold demonstrating that PbPhLP-3 possesses at least one redox-active cysteine. No significant reduction of insulin by DTT was observed in the absence of enzyme (Fig 5C).

### PbPhLP-3 is reduced by the Trx-system

**Fig 6:**
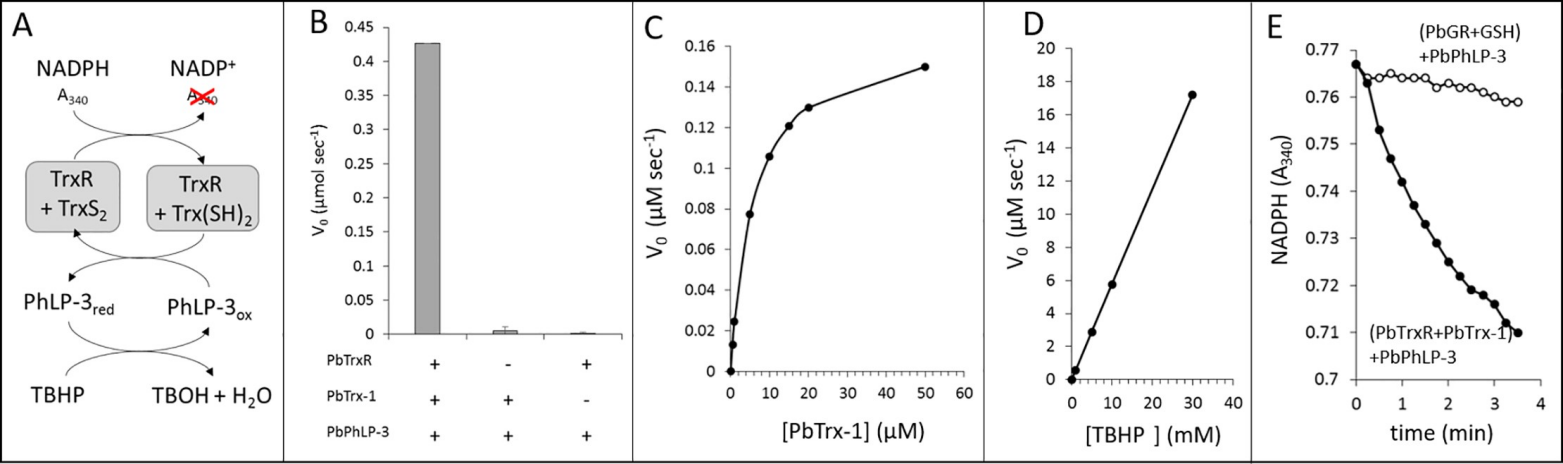
PbPhLP-3 reacts with Trx-system in a Michaelis-Menten type kinetics. **A**. Outline of the Trx reduction assay which measures the oxidation rate of NADPH at 340 nm. **B**. Redox activity was detected only in the presence of all three components. **C**. Graphing initial velocities as a function of varying PbTrx-1 concentrations shows Michaelis-Menten type kinetics between PbTrx-1 and PbPhLP-3. **D**. Initial velocities as a function of TBHP concentrations show a linear relationship between PbPhLP-3 and TBHP. No significant NADPH oxidation was observed in the absence of either PhLP-3 or TBHP OH. **E**. NADPH oxidation assay over time show that PbPhLP-3 is reduced by the Trx system (closed circles) but not by the GSH system (open circles). The slope observed in the PbGR/GSH reaction is comparable to the auto-oxidation rate of NADPH (not shown).

In biological systems, including the malaria parasite, some members of the Trx-superfamily serve as target-proteins for the cellular Trx-system [36, 37]. To assess whether PbPhLP-3 could be a potential target for the Trx-system we conducted *in vitro* Trx-reduction assays utilizing the endogenous *P*. *berghei* Trx-system, which consists of the *P*. *berghei* thioredoxin reductase (PbTrxR) and PbTrx-1 as previously described [38]. PbPhLP-3 was added to a reaction containing NADPH, and the *P*. *berghei* Trx-system. Oxidation of NADPH was followed in a spectrophotometer at 340 nm (Fig 6A). NADPH oxidation was observed in the presence of PbPhLP-3 at an initial rate of 6.5 μM min^-1^ indicating electron flow from the Trx-system to PbPhLP-3 (S1A Fig). Following this result, we tested whether PbPhLP-3 accepts electrons from the flavoenzyme PbTrxR or PbTrx-1, respectively. The experiment was repeated in the absence of PbTrx-1 and no significant NADPH oxidation was observed. Subsequent addition of PbTrx-1 to the assay resulted in rapid NADPH oxidation signifying that PbPhLP-3 can serve as target protein for PbTrx-1 and that it is not a substrate for PbTrxR (Figs 6B and S3A Fig).

We next determined basic kinetic parameters for the redox reaction between PbTrx-1 and PbPhLP-3 (Fig 6C). Measurement of initial velocities at varying PbTrx-1 concentrations (0.5–50 μM) resulted in a hyperbolic curve characteristic for Michaelis-Menten type saturation kinetics (Fig 6C and Table 2). The K_M_ for PbTrx-1was determined to be 5.82 μM with a k_cat_ of 1.0 min^-1^. The rate constant for this reaction was calculated as 1.7 x 10^5^ M^-1^ min^-1^.

**Table 2:** Comparison between the kinetic parameters of PbPhLP-3 and HsPhLP-3.

	*PbPhLP-3*	*HsPhLP-3*
**Length (AA)**	193	226
**M_w_ (kDa)**	22.9	26.5
**IP**	6.5	5.4
**K_M_ [μM]**	5.82	6.53
**V_max_ [μM min^-1^]**	10.05	12.61
**k_cat_ [min^-1^]**	1.00	1.26
**k_cat_/K_M_ [M^-1^ min^-1^]**	1.7 x 10^5^	1.9 x 10^5^

As some Trx-like proteins of *Plasmodium*, such as *thioredoxin-dependent peroxidase-1* (TPx-1), exhibit antioxidant activity [39], we next determined whether recombinant PbPhLP-3 has the capacity to reduce the reactive oxygen compound *tert butyl hydroperoxide* (TBHP). TBHP was added to the Trx-reduction assay and reduction of the compound was observed in the presence of PbPhLP-3, demonstrating that PbPhLP-3 has the capacity to reduce TBHP (Fig 6D). No significant TBHP reduction was observed in the absence of PbPhLP-3. Increasing TBHP concentrations resulted in a corresponding linear increase of PbPhLP-3 activity suggesting a non-enzymatic reaction mechanism. It should be noted, that significant NADPH oxidation was only detectable at TBHP concentrations in the millimolar range. In comparison, the antioxidant protein TPx-1 in *P*. *falciparum* effectively reduces TBHP in the micromolar range [7].

The oxidoreductase *glutathione reductase* (GR) and the tripeptide *glutathione* (GSH), constitutes the cellular GSH-system, a second major thiol-based redox system present in most organisms, including *Plasmodium* [5]. Cross talk between the Trx- and the GSH-systems occurs frequently [4, 40] and we therefore assessed whether PbPhLP-3 could also serve as target for the GSH system. The thiol-reduction assay was repeated utilizing recombinant PbGR/GSH in place of PbTrxR/PbTrx-1 (Fig 6E). No significant oxidation of NADPH in the presence or absence of PbPhLP-3 was observed indicating that PbPhLP-3 is not a target for the GSH-system.

### Hypothetical 3D model and site-directed mutagenesis

**Fig 7:**
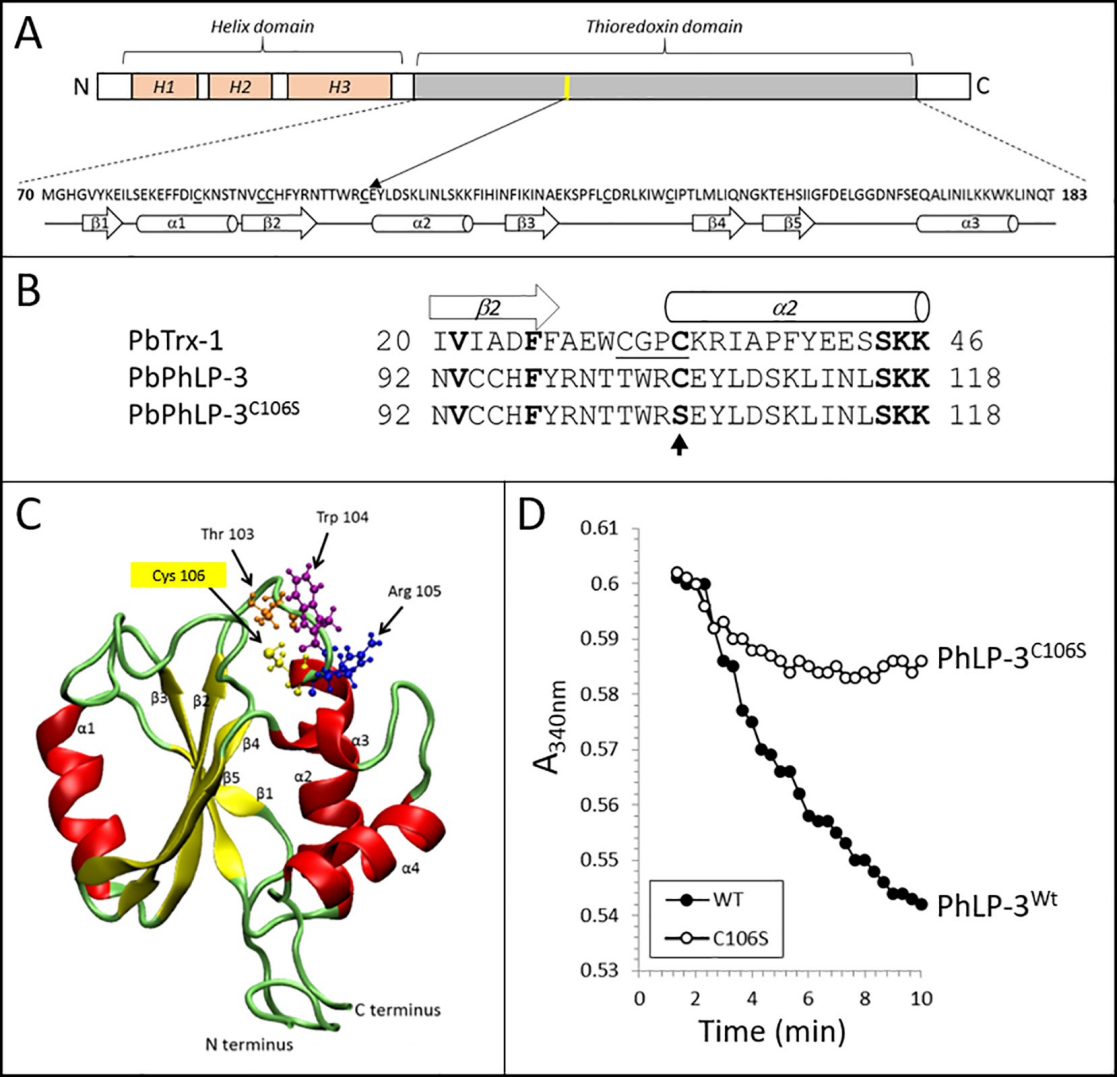
Sequence alignment, 3D modeling and site-directed mutagenesis locates redoxactive cysteine in PbPhLP-3. **A**. Schematics of PbPhLP-3 showing the predicted domains. The sequence of the thioredoxin domain is shown with all cysteines underlined. The yellow bar indicates the putative redox active cysteine. Secondary structure prediction within the thioredoxin domain are also shown. **B**. Alignment of the PbTrx-1 active site (PDB ID: 2MMN; [42] with the corresponding PbPhLP-3 wt and mutagenized sequences, respectively. The SDM site is indicated by an arrow. **C**. Hypothetical model of the Trx-domain of PbPhLP-3. The recently crystallized Trx-domain of the human phosducin-like protein 2 (hPDCL2) (PDB ID: 3EVI) served as template [41]. Amino acids of a hypothetical active site are indicated as ball-and-stick representation. **D**. Trx-reduction assays comparing redox activities of PbPhLP-3^wt^ and PbPhLP-3^C106S^.

This is the first report demonstrating redox activity of a PhLP despite the lack of a characteristic active site motif, such as [CXXC]. Examination of the primary sequence shows that PbPhLP-3 contains six cysteines within its predicted Trx-domain (Fig 7A). Alignment of the Trx-domains of PbTrx-1 with that of PbPhLP-3 shows that C106 of PbPhLP-3 aligns with the redox active C33 of PbTrx-1 (Fig 7B). A hypothetical 3D-model using the recently resolved crystal structure of the Trx-domain of *human phosducin-like protein 2* (hPDCL2) [41]) as a template shows the characteristic Trx-fold, consisting of a central twisted 5-stranded β-pleated sheet sandwiched by four α-helices (Fig 7C). Notably, the model placed C106 in a position similar to that of C33 in the active site motif of *Plasmodium* Trx-1 (PDB ID#2MMN; [42]). We therefore hypothesized that C106 may facilitate the observed redox activity of PbPhLP-3. To test this we performed site-directed mutagenesis (Fig 7B) and replaced C106 with serine. The resulting mutated recombinant protein (PbPhLP-3^C106S^) expressed and purified as described for the wild-type protein (PbPhPL-3^wt^). Repeating the insulin-reduction assay with PbPhLP-3wt or PbPhLP-3C106S resulted in a significantly reduced insulin reduction rate in the presense of the mutagenized PbPhLP-3 (S3B Fig). Subsequent testing of PbPhLP-3^C106C^ and PbPhPL-3^wt^ in the Trx-reduction assay also showed significantly reduced redox-activity with the mutant PhLP-3^C106C^ (Fig 7D) supporting our hypothesis that C106 plays a significant role in the redox activity of PbPhLP-3. Notably, this redox active cysteine in the protozoan protein is conserved throughout the PhLP-3 family (see Fig 4A).

### Redox activity is conserved in human PhLP-3

**Fig 8:**
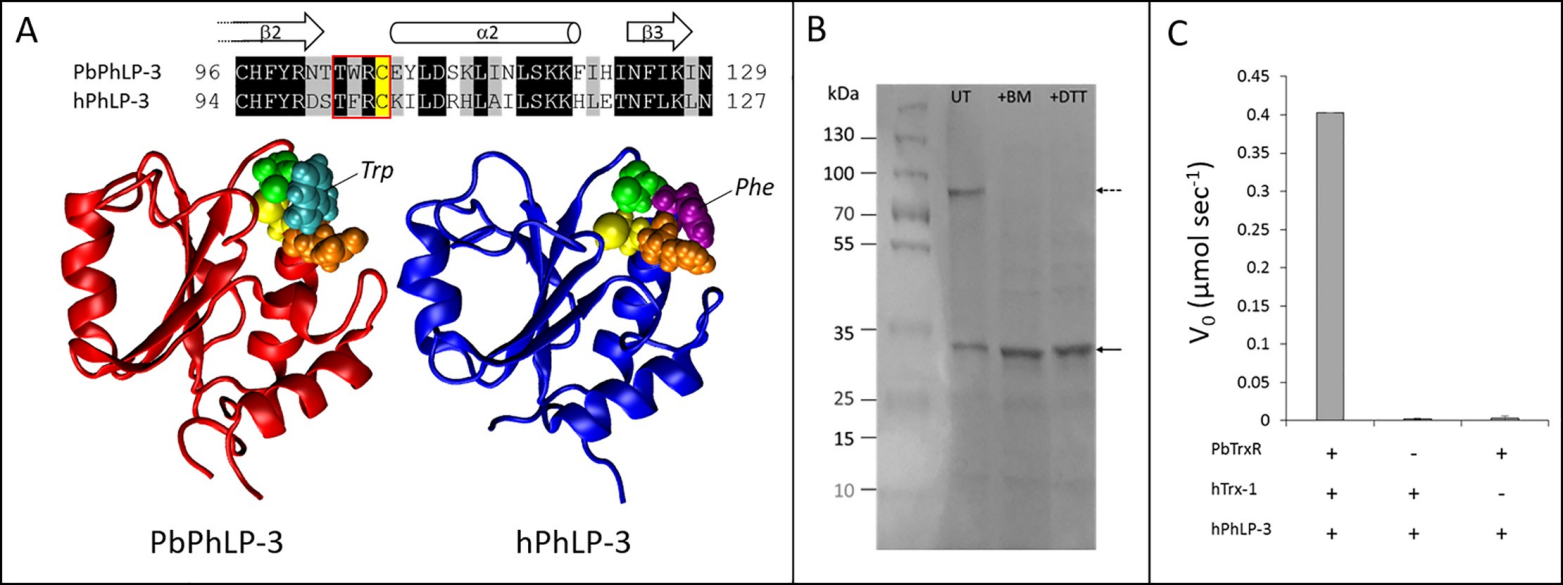
Redox activity is conserved between *Plasmodium* and human PhLP-3. **A**. Sequence alignment around the putative active site (red box) indicates high conservation, the redoxactive cysteine is highlighted in yellow. Comparison between the hypothetical models of the *Plasmodium* (red) and the human (blue) PhLP-3 Trx-domains show high structural conservation. Putative active site residues are depicted in Van der Waals mode threonine (green), tryptophan (turquoise), phenylalanine (purple), arginine (orange), cysteine (yellow). **B**. SDS Page of PbPhLP-3 purification over a Ni-NTA column. Samples remained either untreated (UT = untreated) or were pretreated with reducing agents 2-mercaptoethanol (BM) or dithiothretiol (DTT). Solid arrow indicates the size of recombinant HsPhLP-3 protein incl. HIS-tag. The distinct band in the UT lane (dashed arrows) designates a potential multimer of HsPhLP-3. **C**. Thioredoxin reduction assays show that activity was detectable only in the presence of all three components.

The human and the protozoan PhLP-3 share over 50% sequence identity in the Trx-domain (Fig 4B), which includes the cysteine identified as redox active in *P*. *berghei*. Amino acid alignment indicates that the [TWRC] motif identified in PbPhLP-3 is 75% conserved in the human homolog with tryptophan (W) in the protozoan sequence replaced by phenylalanine (F) in the human sequence, resulting in a [TFRC] site (Fig 8A). This raised the hypothesis that HsPhLP-3 may also exhibit redox activity. To test this, we cloned, expressed, and purified HsPhLP-3 following the same protocol described above for PbPhLP-3. HsPhLP-3 is located on chromosome 2 in the human genome and codes for a 226 amino acid protein with a calculated molecular mass of 27 kDa [15]. The size difference of 5 kDa to the protozoan PhLP-3 is due to a longer C-terminus (30 amino acids) (see Fig 3A and 3B). An SDS gel of the purified HIS-tagged HsPhLP-3 revealed two prominent bands under non-reducing conditions, one at about 30 kDa and a second at ~ 80 kDa (Fig 8B). The smaller band corresponded to the HIS-tagged HsPhLP-3. Running the sample in the presence of *DTT* or *2-mercaptoethanol* caused the 80 kDa band to collapse to the 30 kDa, band suggesting that the larger band represents a thiol-based multimer of recombinant HsPhLP-3.

Following confirmation that recombinant HsPhLP-3 is redox active in the insulin assay (S3A Fig) we repeated the Trx-reduction assay as described earlier with human Trx-1 (HsTrx-1) replacing PbTrx-1 and HsPhLP-3 replacing PbPhLP-3, respectively (Fig 8C). We used PbTrxR as oxidoreductase, since effective reduction of HsTrx-1 by *Plasmodium* TrxR had been described previously [4]. Addition of recombinant HsPhLP-3 to a NADPH/PbTrxR/HsTrx-1 reaction resulted in rapid oxidation of NADPH comparable to the data we collected for PbPhLP-3, confirming redox activity of HsPhLP-3 (Fig 8C). In a follow-up assay, we reversed the order of the substrates by starting with an NADPH/HsPhLP-3 mix. The addition of PbTrxR did not result in significant NADPH oxidation. Subsequent addition of hTrx-1 caused a rapid drop in absorption verifying electron flow from NADPH to PbTrxR to HsTrx-1 to HsPhLP-3 (S3B Fig). We subsequently determined the K_M_ for the reaction between HsTrx-1 and HsPhLP-3 to be 6.53 μM with a k_cat_ of 1.26 min^-1^ and a rate constant of 1.9 x 10^5^ M^-1^ min^-1^ (Table 2). These data were consistent with those detected in the PbTrx-1/PbPhLP-3 assay and demonstrate not only that redox activity is conserved between protozoan and human PhLP-3 but also that either protein can serve as a Trx-1 target protein.

Based on our results we propose the following general reaction scheme for PhLP-3:

1. NADPH + H^+^ + TrxS_2_ ⇆ NADP^+^ + Trx(SH)_2_ (*TrxR catalyzed*)
2. Trx(SH)_2_ + PhLP-3_ox_ ⇆ TrxS_2_ + PhLP-3_red_

## Discussion

PhLPs are small Trx-domain containing proteins that are highly conserved in eukaryotes from yeast to human [10, 11, 28, 43] where they interact with trimeric G-proteins [12] and function as co-chaperones in the cytosolic protein folding machinery [43]. We recently identified three novel Trx-like genes in the *Plasmodium* genome as members of the *phosducin-like family of proteins* (PhLPs) [10].

In this study, we characterize the first PhLP of a protozoan organism, the rodent malaria parasite *P*. *berghei*. The complex life cycle of *Plasmodium* spans two very different host organisms and can be generally divided into blood stages, which take place in the mammalian host, and mosquito stages, which occur in the mosquito. Gene expression profiles for the parasite can provide information about the significance of a particular gene for a particular developmental stage. Our transcription data does not indicate any stage-specific expression for *pbphlp-3*. We observed constitutive, low-level expression in the blood- as well as in mosquito stages of *P*. *berghei*. Furthermore, the expression profiles of pbphlp-3 in parasites from the mosquito blood meal and from parasite cultures are comparable. This suggests that *pbphlp-3* expression is largely independent of environmental changes, unlike other Trx-related genes, such as *1-cys prx* and *tpx-1* [21]. Data from several *Plasmodium* transcriptome datasets complement these observations and confirm continuous low-level expression of the *pbphlp-3* in all asexual blood stages [16]. This trend continues during parasite development in the mosquito bloodmeal. It is worth noting that spectra of PbPhLP-3 were detected in proteomics data sets of *Plasmodium* sporozoites [19, 44] supporting our hypothesis that the protein is expressed throughout the entire parasite life cycle.

Our inability to generate *pbphlp-3* deficient *P*. *berghei* parasites suggests that *pbphlp-3* plays an essential role in this protozoan organism. This is at least the case for the blood stages of the parasite as those are the principal developmental stages on which genetic manipulation of *Plasmodium* is typically performed [26]. Notably, the database for genetically modified rodent malaria parasites (RMgmDB), which contains information on successful gene disruption in *P*. *berghei*, has no record on a *pbphlp-3* knock out parasite [20]. It does, however, contain an entry describing the successful disruption of *pbphlp-2*, which does not seem to have any significant effect on blood stage *P*. *berghei* [20].

Low-level *phlp* expression and lack of response to external stimuli have been reported previously in other unicellular organisms, including yeast (*plp1*). Disruption of the *plp1* locus in *Saccharomyces* (*plp1Δ*) does not have any effect on the organism [45]. A similar result was reported for the *phlp-3* gene in the ameba *Dictyostelium* [13]. Interestingly, in contrast to the results reported here for *pbphlp-3* disruption of the *phlp-2* gene in either of these organisms results in a lethal phenotype [13, 45].

Recombinant PbPhLP-3 is the first member of the PhLP family shown to exhibit redox activity. It was assumed that PhLPs are not redox active due to the lack of a discernable [CXXC] active site motif that is characteristic for many redox active Trx and Trx-like proteins [2, 14, 28]. Furthermore, PbPhLP-3 exhibits thiol-based redox activity facilitated in part by a cysteine located within the highly conserved Trx-domain. Sequence alignment of PbPhLP-3 with the canonical PbTrx-1 aligns the PbTrx-1 [CGPC] active site motif with the sequence [TWRC] in PbPhLP-3 [10], which includes the redox active cysteine. It is possible that a second, distant cysteine is involved in the redox mechanism. The hypothetical 3D model of PhLP-3 and the VMD graphics analysis suggests that C137 could be surface exposed and in a close enough proximity to C106 to allow for the formation of a disulfide bridge (S6 Fig). Although this cysteine seems to be conserved in the mammalian homologues it does not occur in any of the other analyzed species. We are currently investigating their potential roles in the redox mechanism. The possible involvement of the other amino acids in the [TWRC] sequence has yet to be investigated. Alternatively, a monothiol mechanism could be employed by PhLP-3s as has been described for other members of the Trx-superfamily, e.g. certain glutaredoxins [46].

**Fig 9:**
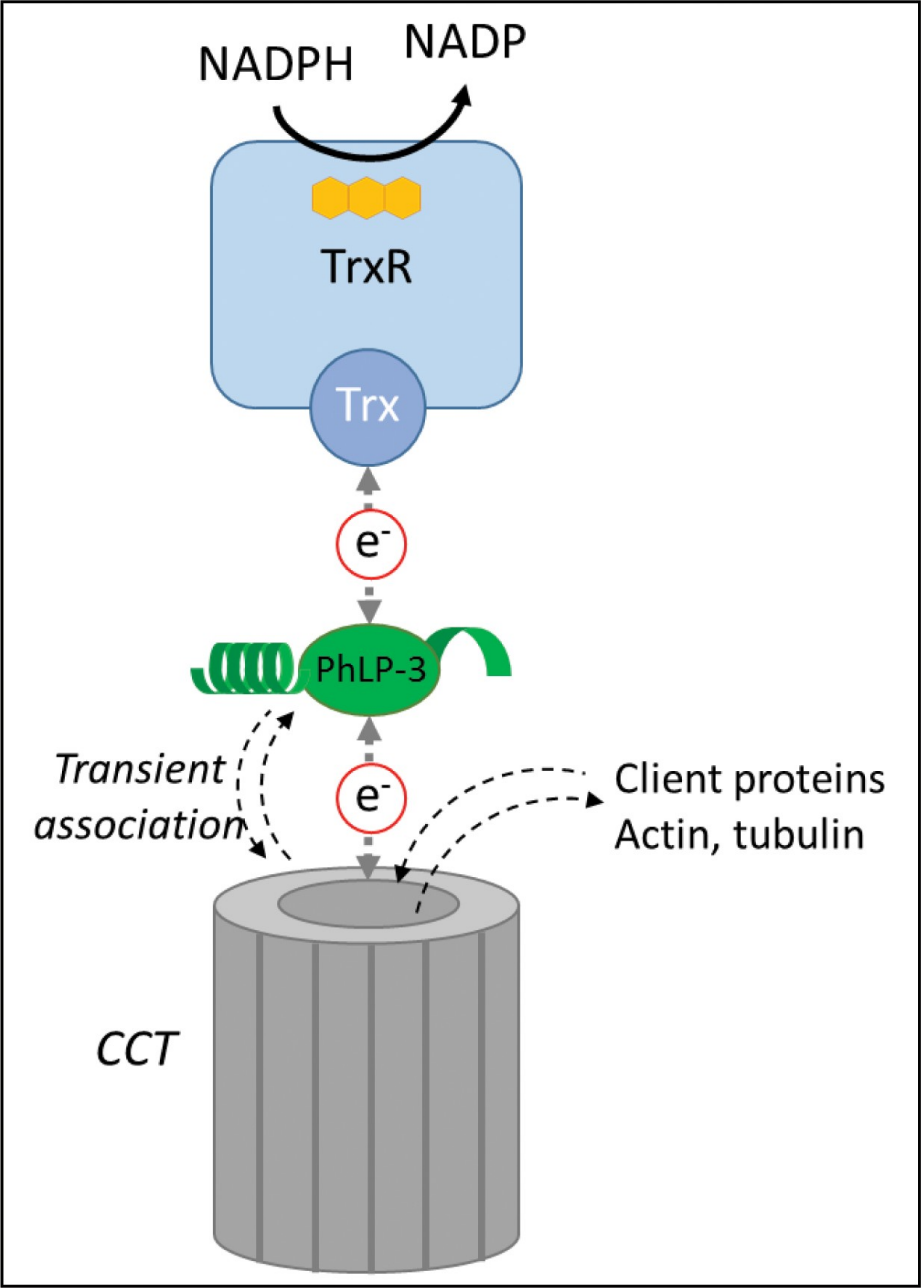
Model showing PhLP-3 as a possible link between the thioredoxin system and the CCT.

PbPhLP-3 and HsPhLP-3 are reduced by the Trx-system *in vitro* and exhibit enzyme kinetics similar to those of other recently characterized Trx-like proteins in *Plasmodium* [7, 38, 47]. This interaction raises the possibility of PhLP-3 being a Trx-target protein *in vivo*. Supporting this hypothesis is the lack of activity with the glutathione (GHS) system, which indicates a certain degree of specificty of PbPhLP-3 for Trx-1. It is, however, possible that the GSH-dependent *glutaredoxin* (Grx), a Trx-1 relative, has the capacity of reducing PhLP-3 as has been reported for the Trx-like *antioxidant protein* (AOP) in *Plasmodium* [7, 48]. AOP, however, is not reduced by the Trx-system. Two studies investigated potential Trx-target proteins in *Plasmodium* using an affinity pull-down approach [36, 37]. Neither of the reports detected PhLPs in their assays. This may be due to low cellular PhLP expression levels, or the fact that PhLP may be complexed with a cellular chaperonin (see below). Nevertheless, the activity with the Trx-system introduces the means for PhLP-3 to receive reducing equivalents and thus may represent a link with the cellular redox system (Fig 9).

Redox active Trx and Tlps have been implemented in antioxidant activity against endogenous and exogenous reactive oxygen (ROS) and reactive nitrogen species (RNS) [49]. Although both PhLP-3 proteins investigated here reduce the oxidative compound TBHP, their efficiencies were several orders of magnitude lower than that of known antioxidant Tlps, such as TPx-1 [7]. The fact that it requires TBHP concentrations in the millimolar range to detect a significant flow of electrons may reflect a low substrate affinity or low enzyme activity by PhLP-3. It should be noted that other ROS, such as hydroxyl radicals, hydrogen peroxide or nitric oxide compounds have not yet been tested with PhLP-3 and a higher activity with these compounds cannot be excluded. However, considering the low-level *pbphlp-3* expression *in vivo* and the absence of upregulation in the presence of exogenous stresses in the mosquito midgut we believe it unlikely for PbPhLP-3 to play a role as an effective antioxidant defense enzyme in the parasite.

Mammalian PhLPs interact with the *Chaperonin Containing TCP-1* (CCT) complex [50, 51], a eukaryotic cytosolic chaperonin central to the folding of actin and tubulin [52]. Recently, the CCT for the human parasite *P*. *falciparum* was isolated and shown to be involved in actin folding [53]. A significant role of PbPhLP-3 in the fundamental process of actin and/or tubulin folding could explain the lack of viable parasites in the knock-out attempts. Supporting this hypothesis are observations that show knockdown of *phlp-3* in higher eukaryotes causes severe phenotypes, primarily affecting cytoskeletal architecture and function [28, 43, 54]. Similarly, overexpression of mammalian *phlp-3* in transgenic Chinese Hamster Ovary cells results in severe structural defects in monomeric tubulin and actin proteins as well as in microtubules and microfilaments [50, 54, 55]. Consequently, a role for PhLP-3 as co-chaperone for actin and tubulin in higher eukaryotes has been proposed [15, 56].

How would redox activity of PhLP-3 fit into the concept of a co-chaperone? Structure analysis of a rat PhLP-3/CCT complex shows that monomeric PhLP interacts with the apical portion of the barrel-shaped CCT complex [51] (Fig 9). The N-terminal helix domain as well as the C-terminal region of PhLP are required for binding to the CCT complex, with the Trx-domain spanning the substrate-binding cavity [15, 51]. While the N-terminal helix domain as well as the C-terminus are essential for PhLP binding to CCT, the function of the highly conserved Trx-domain has not been investigated [14, 43, 51]. HsPhLP-3 forms a ternary complex with CCT substrates actin and tubulin, suggesting an active role in the protein folding process [15]. PhLP-3 could actively be involved in the folding process. Several members of the Trx-superfamily that exhibit thiol-based redox reactions function as chaperones. Most prominent are the *Protein Disulfide Isomerases* (PDI) in the endoplasmic reticulum (ER) of eukaryotic cells [57] including the Malaria parasite [58] PDIs facilitate client protein folding via the temporary formation of disulfide bridges, a process known as *redox-assisted protein folding*[59]. PhLP-3 may function in a similar fashion. Inter- or intramolecular disulfide-bridges formed during the folding process may be reduced by PhLP-3, which in turn receives electrons from the Trx system or related redox active protein(s). Alternatively, the redox state of PhLP-3 may serve to regulate its interactions with the CCT complex and thus entry or exit of client proteins (Fig 9). Transient association of PhLP with the CCT complex has been hypothesized given that other proteins, such as the prefoldin, compete for the same binding site on the CCT [43, 60]. PhLP-3 may thus function as “gatekeeper” to regulate entry and exit of CCT client proteins, specifically actin and tubulin. We are currently investigating the *in vivo* relevance of the PhLP-3 redox activity as we hypothesize that PhLP-3 functions as a link between the cellular redox system and the protein folding machinery.

## Conclusion

The characterization of the first protozoan PhLP provides new insights into the functional capabilities of PhLP-3. It will be important to further investigate the significance of the PhLP-3 redox-activity as it is likely to be fundamentally important for eukaryotic cell biology.

Moreover, the effective reduction of PhLP-3 by the Trx system could represent a link between the redox regulatory system and the cytosolic protein folding and regulation machinery. Ongoing investigations into the structure-function relationship of PhLPs will give further insights into the potential protein folding mechanism and regulation of cytoskeletal elements in eukaryotic cells in general [43] and in protozoan parasite in particular[53].

## Material and methods

### Ethics statement

All experimental protocols involving mice, specifically ketamine-induced anesthesia in mice for mosquito feeds, were approved by the Institutional Animal Care and Use Committee (IACUC) of Loyola University Chicago (Protocol#1429), which follows the National Institutes of Health (NIH) guidelines for animal housing and care.

### Parasite maintenance and mosquito infections

*Plasmodium berghei* parasites (ANKA 2.34; originated from the Johns Hopkins Malaria Research Institute Parasite Core facility [61]) were maintained in female CF-1 mice (Charles River) for a maximum of four serial passages and regularly passed through *Anopheles stephensi* mosquitoes. *A*. *stephensi* mosquitoes (Sind-Kasur Nijmegen strain) originated from the Malaria research group at Radboud University, Nijmegen, Netherlands [62]. Mosquitoes were reared under standard conditions (30°C, 80% RH, 12 hrs. light-dark cycle, 5% sucrose solution). Female mosquitoes (5–10 days post emergence) were used in all experiments. Mosquitoes were fed on *P*. *berghei* infected mice (10%). Exflagellation (2-4/20x) of parasites was determined prior to feeding to ascertain parasite maturity. Blood-fed mosquitoes were [62]maintained at 21°C and 80% RH to allow for parasite development. Midguts of 40–50 mosquitoes were dissected per experimental time point and transferred to Tri-Reagent RT (MRC gene) for total RNA extraction. Following each feed, we maintained 20 mosquitoes to verify infection by counting oocysts following mercurochrome stain of dissected midguts nine days post-infectious blood meal.

### RNA extraction and quantitative real-time RT-PCR (RT-qPCR)

Total RNA was extracted from dissected mosquito midguts using Tri-Reagent RT (MRC) following manufacturer’s instructions. Isolated RNA was treated with DNAse I (Ambion) and subsequently quantified using a nanodrop 2000 (Thermo). RNA-samples were either immediately used for cDNA synthesis or flash frozen and stored at -80°C. cDNA was synthesized from total RNA with the High Capacity RNA-to-cDNA kit (Applied Biosystems) using random hexamer primers. Sequences of target genes for primer design were acquired from Plasmodb (plasmodb.org) (S1 Fig). RT-qPCR was performed on a StepOnePlus machine (Applied Biosystems) using the Fast SYBR Green Master Mix (Applied Biosystems). Each sample was run in triplicates and yielded highly comparable Ct values (cycle threshold). No primer dimers were detected and amplicons exhibited optimal efficiencies. To test specificity all primer pairs were tested on uninfected mouse blood, non-fed and uninfected blood fed mosquitoes. No amplification products were detected. Expression data was subsequently analyzed with the StepOne Software v2.2 (Applied Biosystems) and normalized against the expression of *P*. *berghei* 18s rRNA A-Type, which is an established internal standard for expression analysis in *Plasmodium* mosquito stages [21, 63, 64]. For analysis of time course expression data, the ΔΔC_t_ method was applied using the earliest experimental time point (T1) as reference sample (RQ = 1). The Mann-Whitney U test was conducted on each candidate gene from both mosquito-derived and from culture-derived parasites. Significance was assessed at p<0.1 due to the low sample sizes. The non-parametric Mann-Whitney U test was the appropriate statistical analytical approach to use on this dataset due to the violated assumptions of independence in the data and the low and unbalanced sample sizes in gene expression collected at 3, 6, 12, and 24 hours. Statistical analyses were performed in the R-language environment.

### Knock-out construct, parasite preparation, transfection, and selection strategies

A targeting vector was constructed in plasmid pBS-DHFR. A 516 bp fragment comprising the 5’ flanking sequence and a 348 bp fragment of the 3’ flanking sequence of *pbphlp-3* were amplified from *P*. *berghei* genomic DNA (S2 Fig). The 5’ fragment was inserted using the ApaI and HindIII restriction sites immediately upstream of the dhfr/ts cassette while the 3’ fragment was inserted into the downstream BamH1 and XbaI restriction sites. The dhfr/ts cassette confers resistance to the antiparasitic compound pyrimethamine. Following sequence verification, the completed *pbphlp-3* KO targeting construct was linearized and purified and then transfected into cultured and purified *P*. *berghei* schizonts as described previously in [24, 27]. The attempt to generate a *pbphlp-3* knockout line was repeated three times.

### Cloning, site-directed mutagenesis, expression, and purification of recombinant PbPhLP-3, PhLP-3^C106S^, and HsPhLP-3

Gene-specific primers for *pbphlp-3* (Plasmodb ID: PBANKA_1204800, NCBI GeneID#: 3423045) and *hsphlp-3* (*aliases*: APACD, TXNDC9; Gene ID# 10190) were generated according to sequence information on PlasmoDB and NCBI, respectively (S2 Fig). PCRs were performed using the following conditions: 35 cycles of 95°C for 30 s, 54°C for 1 min, and 63°C for 45 s. Following sequence verification, the *pbphlp-3* cds was cloned into the bacterial pQE30 expression vector (Qiagen) which introduced a 6xHIS tag at the N-terminus of the recombinant protein. The *pbphlp-3* expression plasmid was transformed into E. coli M15 cells (Qiagen). Human PhLP-3 expression using pQE30 did not work. The *hsphlp-3* coding sequence was therefore cloned into the bacterial pRSET-A expression vector (Thermo Fisher), which also provides an N-terminal 6xHIS tag. The resulting plasmid was then transformed into BL21(DE3) expression cells. To generate the mutated *pbphlp-3* we utilized the Phusion Site-Directed Mutagenesis kit (Thermo Fisher). Using two phosphorylated primers, one containing the cysteine to serine mutation, we modified and amplified the entire pQE30-*pbphlp-3* plasmid, which now coded for PhLPC106S. Protein expression was induced (1 mM IPTG) and bacteria were harvested after a 5 hours incubation period at 37°C. Recombinant proteins were purified using Ni-NTA resin (Thermo Scientific). The purity of the recombinant proteins was assessed via SDS-PAGE. Protein concentrations were determined using the Qubit fluorometer (Invitrogen).

### Enzyme assays

All enzymatic assays were carried out in 1 ml volume at 25°C using a Genesys6 UV-Vis spectrophotometer (Thermo Fisher). The *insulin reduction assay* was conducted as described [32]. Enzymatic activity of the recombinant protein was determined by adding varying amounts of purified PbPhLP-3, HsPhLP-3 or PbTrx-1 to a reaction mixture containing bovine insulin (44 μg ml-1) and DTT (1 mM) in a potassium phosphate buffer (100 mM potassium phosphate, 2 mM EDTA, pH 7.4). The reduction of the insulin disulfide bonds was monitored as an absorbance increase over time at 650 nm. The coupled enzymatic assays utilizing thioredoxin reductase/thioredoxin were conducted as previously described [4, 48]. Briefly, oxidation of NADPH was followed as an absorption decrease at 340 nm. All assays were performed at RT in assay buffer containing 100 mM KH_2_PO_4_, 2 mM EDTA, pH 7.4, 200 μM NADPH (ε_340 nm_ = 6.22 mM^-1^cm^-1^), and 20 μM PbTrx-1 (S4 Fig). Each initial reaction was started with PbTrxR and the decrease of absorption at 340 nm was monitored during the linear phase. Initial velocities and kinetic values for each reaction were determined using the VISIONlite (Thermo Fisher) software. Enzyme kinetics were calculated using global curve fit in the Enzyme Kinetics Module of Sigma Plot 12.0.

### Comparative modeling

The hypothetical models of PbPhLP-3 and HsPhLP-3 were generated using Swiss Model (http://swissmodel.expasy.org; [65]). The crystal structure of the Trx-fold domain of the human PhLP-2 (PDB ID: 3EVI; [41]) was selected as the template from the RCSB Protein Data Bank (http://www.rcsb.org/). The models were visualized using the Visual Molecular Dynamics (VMD) molecular graphics program [66].

## Supporting information

S1 Fig PbPhLP-3 translation map and primer positions. (TIF)

S2 Fig Primer sequences. (TIF)

S3 Fig Insulin assays using PbTrx-1, PbPhLP-3, HsPhLP-3, BSA and PbPhLP-3^Wt^, PbPhLP-3^C106S^. Oxidation of insulin over time in the presence of **A.** PbTrx-1, PbPhLP-3, and HsPhLP-3 and **B.** PbPhLP-3^WT^ and PbPhLP-3^C106S^, respectively. BSA was used as negative control.
(TIF)

S4 Fig Thioredoxin-reduction assays determine order of electron transport. The oxidation of NADPH was measured as a decrease in absorption at 340 nm over time. **A**. PbPhLP-3 Reaction 1 (filled circles) was started with a mixture of NADPH and oxidized PbTrx-1. PbTrxR was added (dashed arrow) and the reduction of PbTrx-1 was allowed to come to completion. Oxidized PbPhLP-3 was then added to the mixture and continuous reduction of NADPH was observed. Reaction 2 (open circles) was started with a mixture of NADPH and oxidized PbPhLP-3. PbTrxR was added (dashed arrow) and reduction of NADPH was measured. After 3 min oxidized PbTrx-1 was added and reduction of NADPH was observed. B. The same experimental approach was used with human Trx-1 and HsPhLP-3 in place of PbTrx-1 and PbPhLP-3, respectively.
(TIF)

S5 Fig Conservation of PhLP-3 among murine and human *Plasmodium* species. Multiple sequence alignment (Clustal W) of putative PhLP-3 proteins of *Plasmodium* species *P*. *berghei* (PB), *P*. *chabaudi* (PC), *P*. *falciparum* (PF), *P*. *knowlesi* (PKH), *P*. *vivax* (PVX) and *P*. *yoelii* (PY) (“*” = Identical; “:” = high similarity; “.” = low similarity). The arrow marks the redox active cysteine describe in this work. The table indicates percent identities.
(TIF)

S6 Fig 3D model of PbPhLP-3 showing hypothetical intramolecular disulfide bridge formation with in the thioredoxin domain. The Visual Molecular Dynamics (VMD) molecular graphics program calculated possible intramolecular disulfide bridge formation within PbPhLP-3. Shown is a energetically possible disulfide bridge between C106 and C137.
(TIF)
