# Supplementary figures and images for: Characterization of a protozoan Phosducin-like protein-3 (PhLP-3) reveals conserved redox activity
Source: PLoS One. 2018 Dec 31;13(12):e0209699. doi: 10.1371/journal.pone.0209699 (PMC6312279; doi:10.1371/journal.pone.0209699)

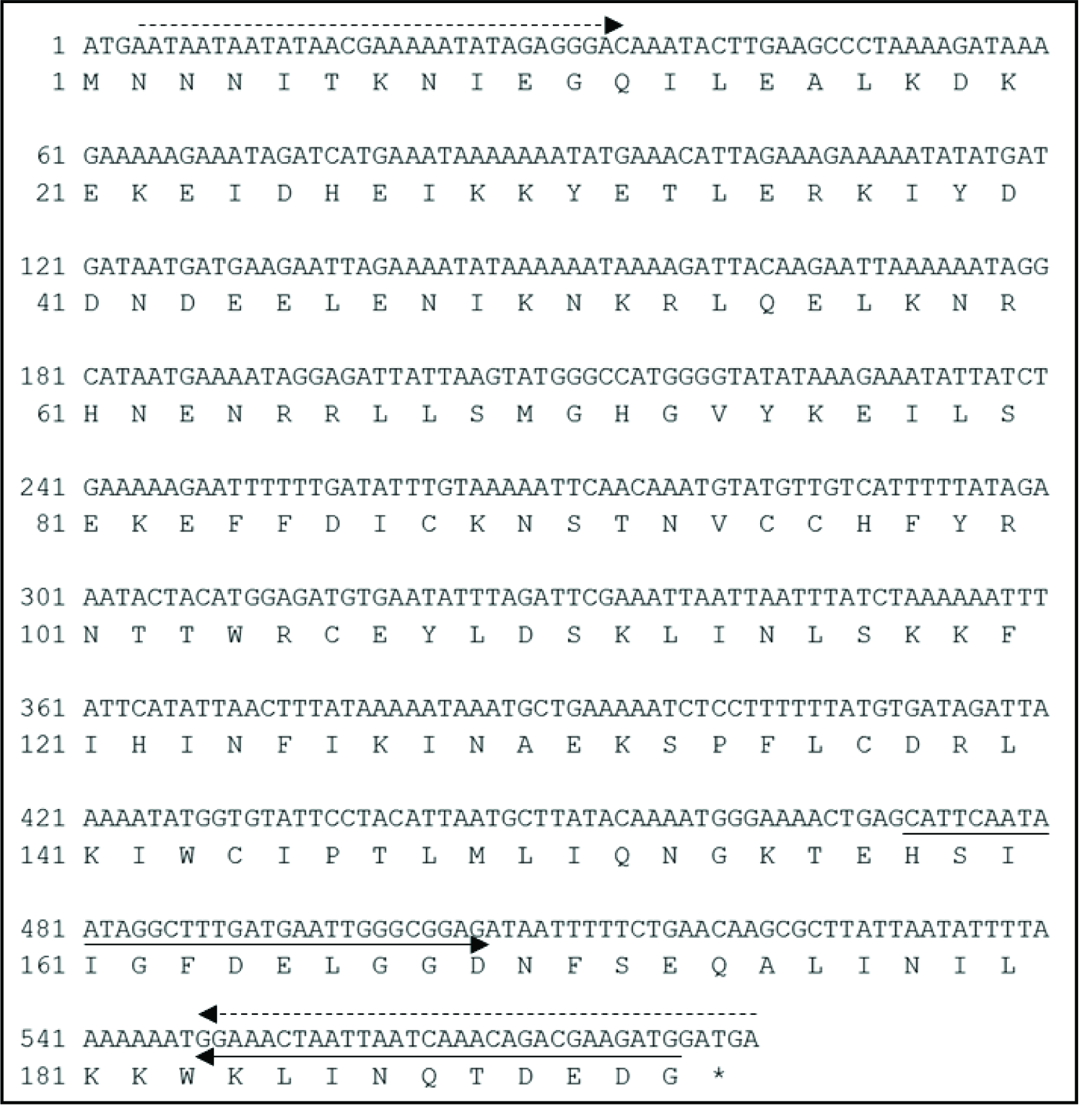

Supplement: S1 Fig — (TIF) [file pone.0209699.s001.tif]

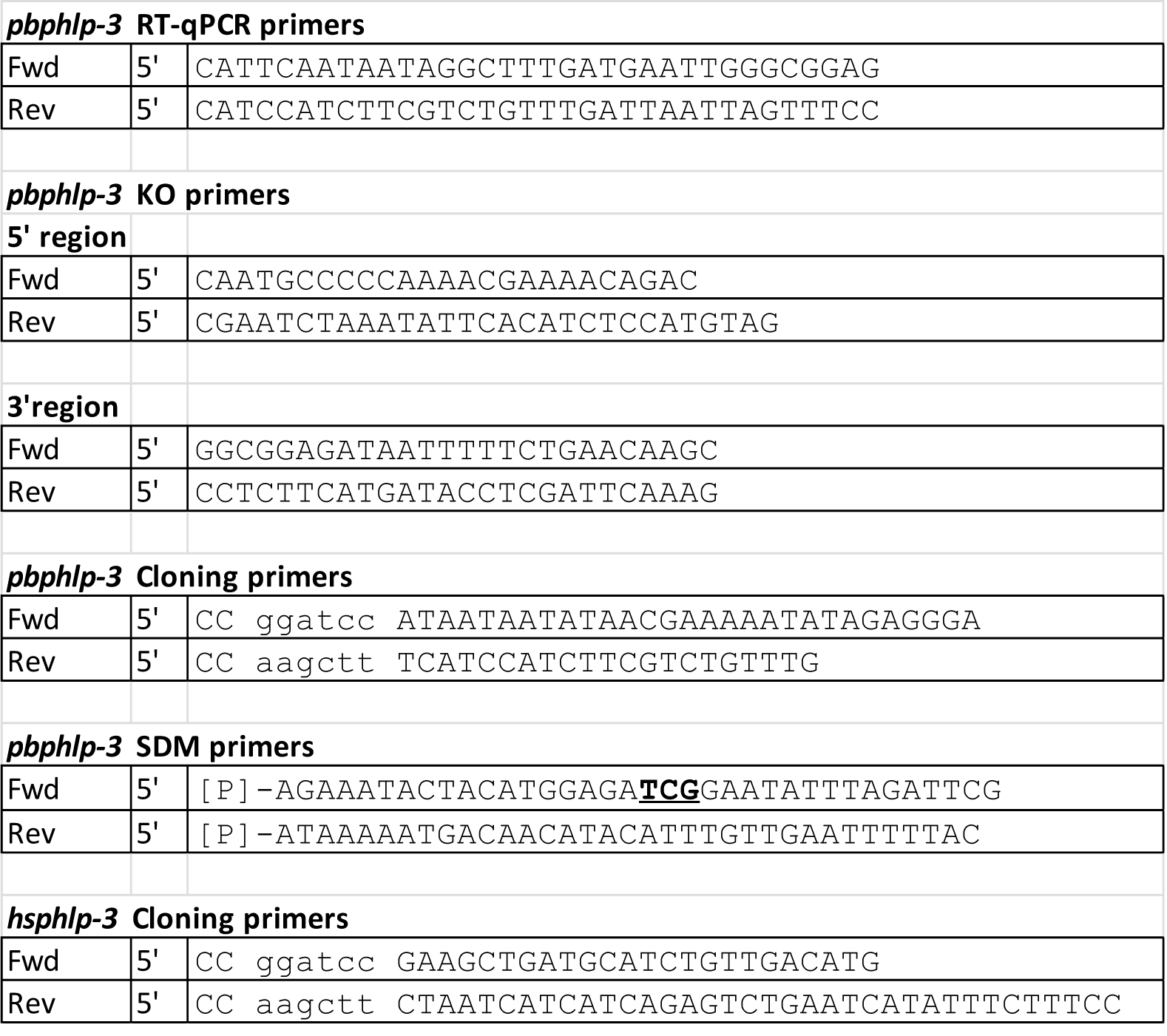

Supplement: S2 Fig — (TIF) [file pone.0209699.s002.tif]

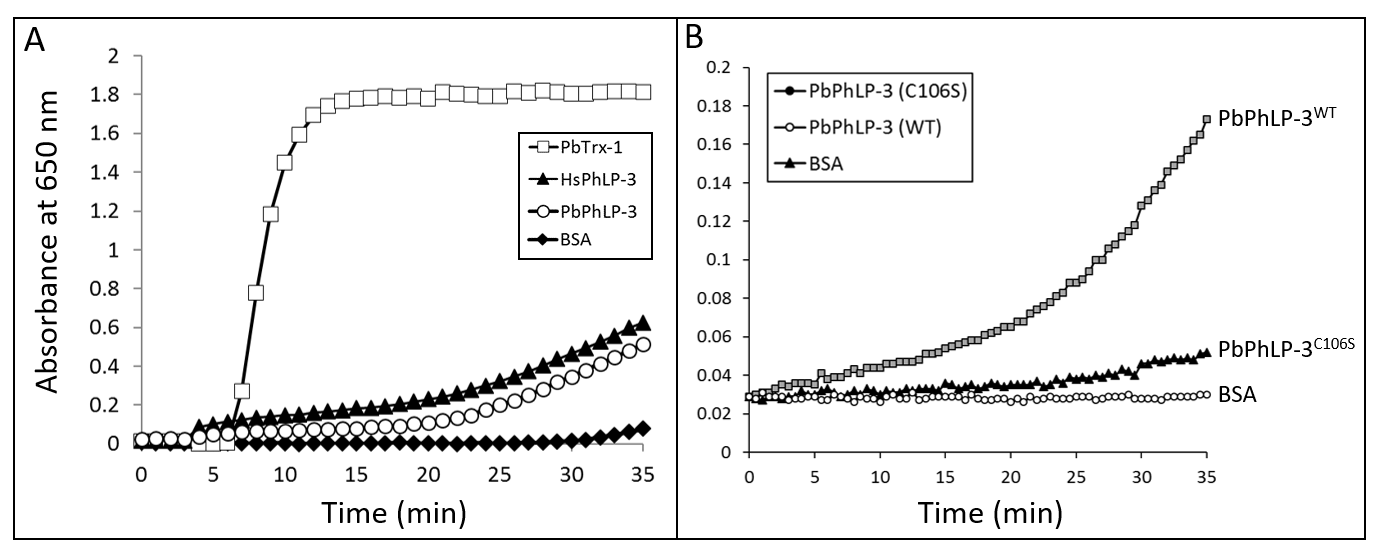

Supplement: S3 Fig — Oxidation of insulin over time in the presence of A. PbTrx-1, PbPhLP-3, and HsPhLP-3 and B. PbPhLP-3WT and PbPhLP-3C106S, respectively. BSA was used as negative control. (TIF) [file pone.0209699.s003.tif]

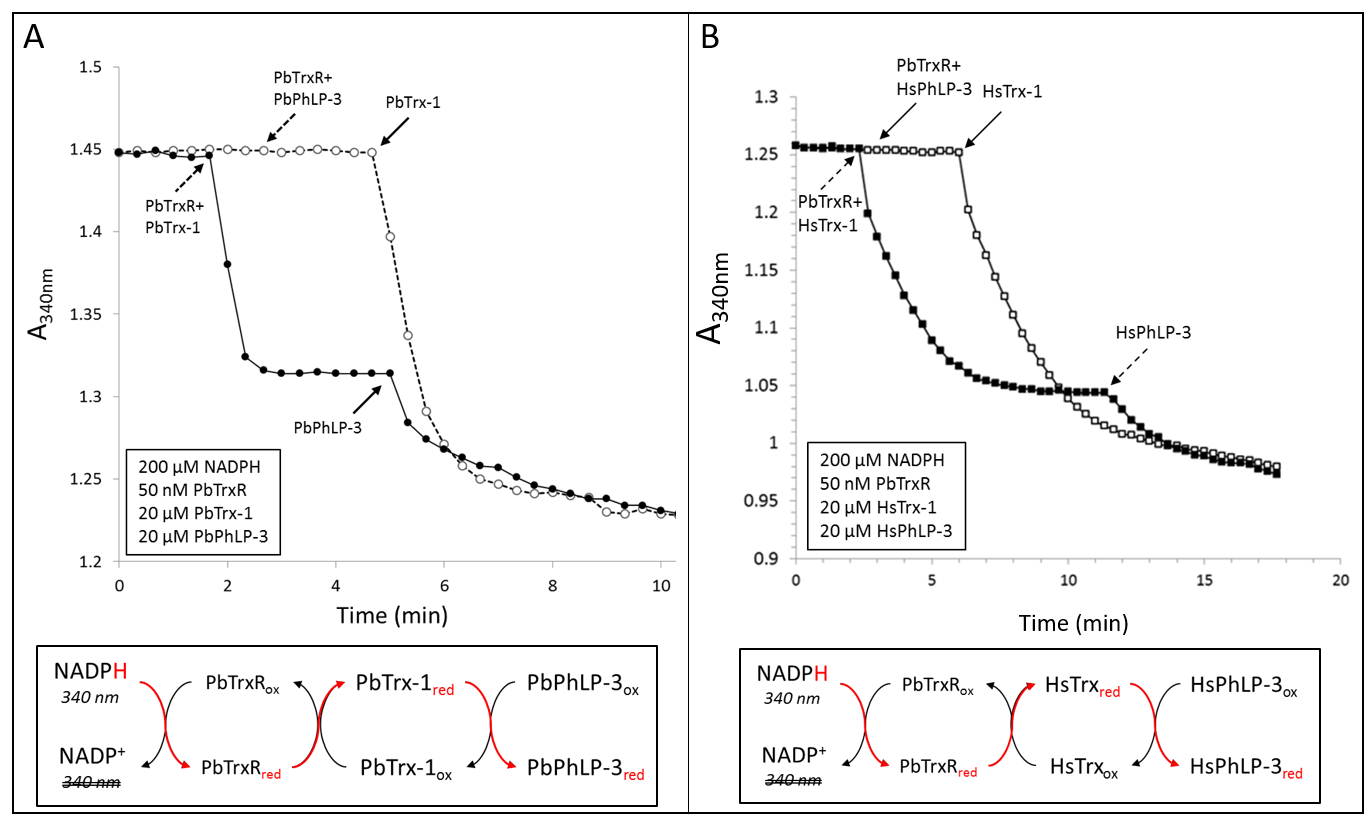

Supplement: S4 Fig — The oxidation of NADPH was measured as a decrease in absorption at 340 nm over time. A. PbPhLP-3 Reaction 1 (filled circles) was started with a mixture of NADPH and oxidized PbTrx-1. PbTrxR was added (dashed arrow) and the reduction of PbTrx-1 was allowed to come to completion. Oxidized PbPhLP-3 was then added to the mixture and continuous reduction of NADPH was observed. Reaction 2 (open circles) was started with a mixture of NADPH and oxidized PbPhLP-3. PbTrxR was added (dashed arrow) and reduction of NADPH was measured. After 3 min oxidized PbTrx-1 was added and reduction of NADPH was observed. B. The same experimental approach was used with human Trx-1 and HsPhLP-3 in place of PbTrx-1 and PbPhLP-3, respectively. (TIF) [file pone.0209699.s004.tif]

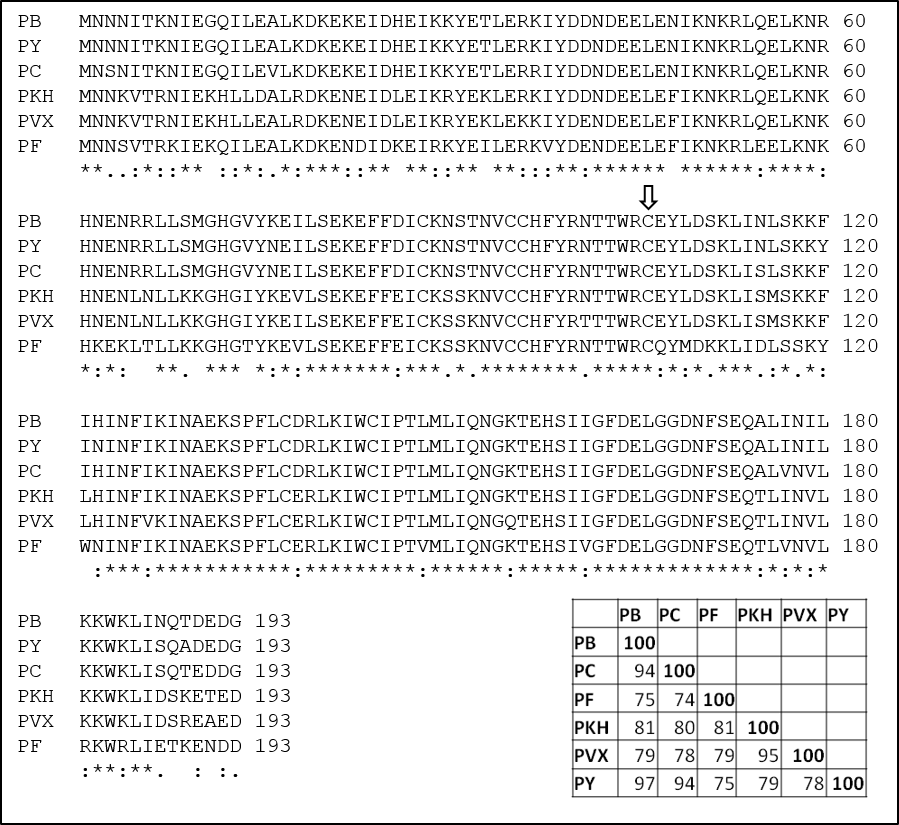

Supplement: S5 Fig — Multiple sequence alignment (Clustal W) of putative PhLP-3 proteins of Plasmodium species P. berghei (PB), P. chabaudi (PC), P. falciparum (PF), P. knowlesi (PKH), P. vivax (PVX) and P. yoelii (PY) (“*” = Identical; “:” = high similarity; “.” = low similarity). The arrow marks the redox active cysteine describe in this work. The table indicates percent identities. (TIF) [file pone.0209699.s005.tif]

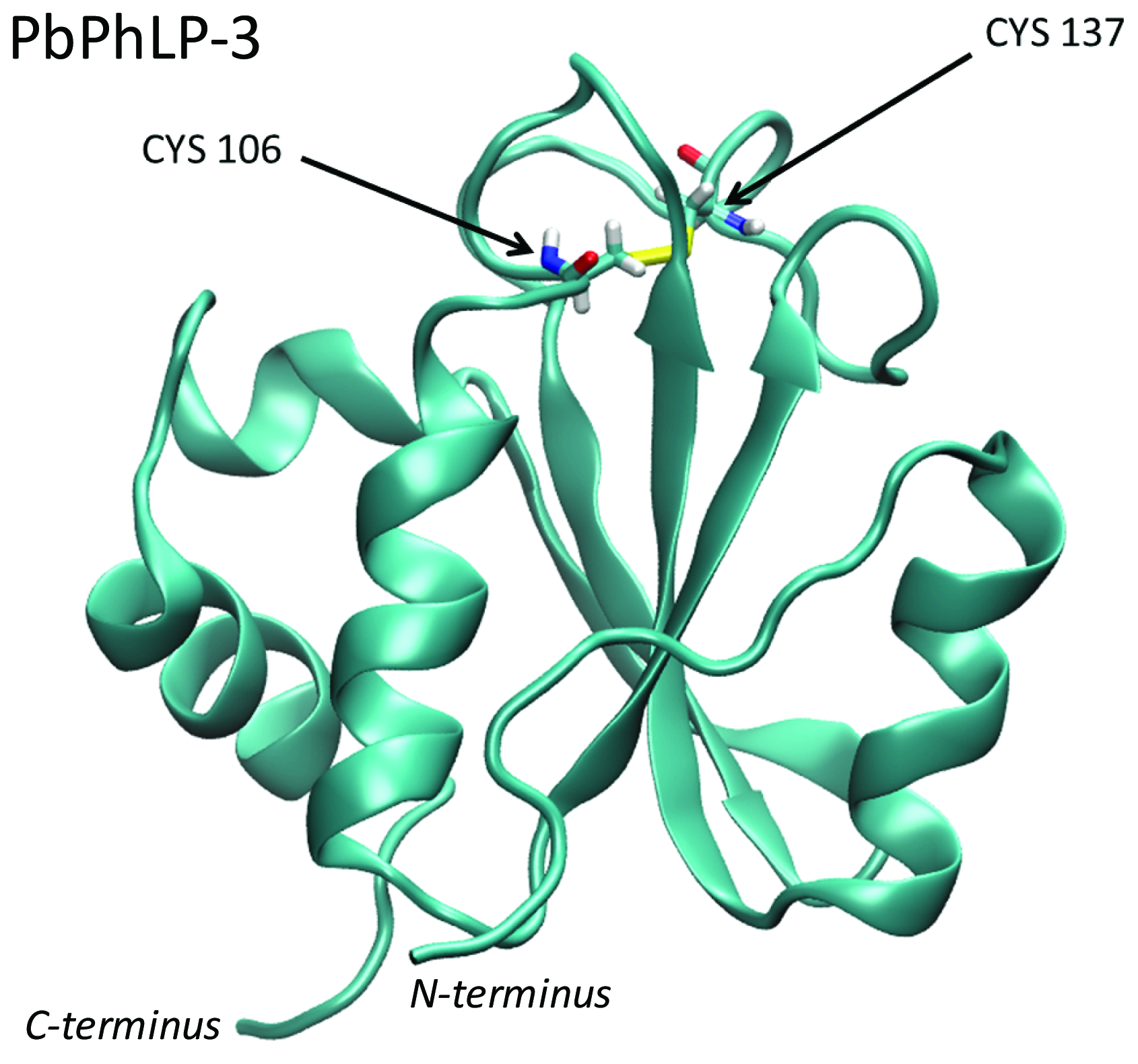

Supplement: S6 Fig — The Visual Molecular Dynamics (VMD) molecular graphics program calculated possible intramolecular disulfide bridge formation within PbPhLP-3. Shown is a energetically possible disulfide bridge between C106 and C137. (TIF) [file pone.0209699.s006.tif]
